# Evaluating the associations of learning environment with student engagement and learning gains among undergraduate students at a medical university in western China

**DOI:** 10.3389/fpubh.2026.1885067

**Published:** 2026-10-19

**Authors:** Zhiwen Mo, Yong Gao, Zhuangyu Wei, Hua Xu

**Affiliations:** 1 Institute of Higher Education Research, Guangxi Medical University, Nanning, Guangxi, China; 2 School of Public Health, Guangxi Medical University, Nanning, Guangxi, China; 3 Department of Clinical Laboratory, The First Affiliated Hospital of Guangxi Medical University, Nanning, Guangxi, China; 4 Key Laboratory of Clinical Laboratory Medicine of Guangxi Medical University, Department of Education of Guangxi Zhuang Autonomous Region, Nanning, Guangxi, China; 5 School of Humanities and Health Management, Guangxi Medical University, Nanning, Guangxi, China; 6 Education Evaluation and Faculty Development Center, Guangxi Medical University, Nanning, Guangxi, China

**Keywords:** learning environment, learning gains, medical education, quality improvement, repeated cross-sectional study, student engagement

## Abstract

**Introduction:**

As quality improvement in medical education increasingly focuses on the learning processes that students actually experience, it is important to clarify which learning-environment domains are most closely associated with student engagement and how engagement relates to learning gains.

**Methods:**

In this pooled repeated cross-sectional study, we analyzed 11,717 student responses from four administrations (2021–2024) of the National College Student Survey among undergraduate students at a medical university in western China. The learning environment was assessed across five domains: classroom teaching quality, facilities and resources, developmental support, interpersonal support, and teaching administration. Student engagement was measured across behavioral, emotional, and cognitive dimensions. Learning gains were assessed across six domains. Hierarchical multiple linear regression, cross-sectional indirect association analysis, year-stratified analyses, and k-means clustering were used to examine associations among perceived learning environment, engagement, and learning gains and to explore heterogeneity among students.

**Results:**

Survey year and background characteristics explained only 1.5–4.0% of the variance in engagement, whereas the five learning-environment domains explained an additional 22.5–27.6%. Classroom teaching quality showed the largest positive associations with behavioral, emotional, and cognitive engagement, which were statistically significant in every survey wave, and developmental support was also positively associated with all three dimensions. Engagement statistically accounted for 31.93% of the cross-sectional association between perceived learning environment and learning gains (total indirect association = 0.234; total association = 0.734). Person-centered analysis identified a convergent-low profile comprising 37.2% of students, whose scores on perceived learning environment, engagement, and learning gains were all below the sample average; background characteristics were only weakly associated with membership in the three profiles (Cramér’s *V* ≤ 0.063).

**Discussion:**

These findings suggest that, in improving educational quality, medical universities should give greater attention to the classroom teaching and developmental support processes that students experience directly in their everyday learning, and should incorporate student engagement and learning experiences into routine, ongoing quality monitoring alongside conventional outcome assessment.

## 1 Introduction

Medical education quality assurance is undergoing a broad international reorientation: the evaluative focus is shifting from institutional inputs, curricular content, and resource provision toward learning processes, learner development, competency attainment, and continuous quality improvement (1, 2). In its 2022 update, the Lancet Commission on the Education of Health Professionals further emphasized that competency-based reform aligned with health system needs represents an important pathway for strengthening health system performance, while also noting that the post-pandemic era has heightened the significance of interprofessional collaboration, digital technology, and pedagogical innovation in medical education reform (2). Concurrently, the World Federation for Medical Education Global Standards for Quality Improvement in Basic Medical Education underscored that quality assurance should extend beyond external evaluation or compliance review to function as a driver of quality improvement; to this end, medical schools are expected to establish quality assurance systems that encompass the full range of educational activities, incorporate feedback from students and other stakeholders, and use relevant evidence to continuously refine educational design and delivery (3).

Against this increasingly outcomes and process-oriented backdrop, student engagement has emerged as an important process-level indicator in health professions education because it reflects the time, effort, and psychological resources students invest in educationally purposeful activities and helps explain how institutional conditions and pedagogical arrangements are translated into learning and developmental outcomes (4, 5). Recent reviews and international guides have further highlighted that student engagement is multidimensional in nature, yet its conceptualization and operationalization remain inconsistent; at the same time, the empirical literature in medical education remains limited in both evidence synthesis and cumulative depth (4, 6).

Theoretically, both engagement scholarship and the input–environment–outcome (I-E-O) framework suggest that the learning environment serves as a critical upstream factor linking institutional conditions to student development, and that its relevance to student development depends not only on the quality of the environment itself but also on the extent to which it elicits students’ active engagement (5, 7). At a finer-grained level, Bronfenbrenner and Morris’s concept of proximal processes proposes that developmental outcomes are shaped primarily by the recurring, progressively complex interactions between the learner and the immediate educational environment, suggesting that not all environmental dimensions may contribute equally to engagement (8, 9). Self-determination theory further suggests that learning environments supporting autonomy, competence, and relatedness are more likely to promote students’ adaptive motivation and engagement (10, 11). In medical education, students’ perceptions of the learning environment have been linked to key outcomes including burnout, empathy, and career regret (12), and more recent work suggests that student engagement may function as an important proximal process connecting environmental conditions to academic and developmental outcomes (4).

However, several gaps limit the translation of existing evidence into targeted quality improvement. First, prior studies have often examined perceived learning environment, student engagement, or outcomes separately, and have rarely tested their joint associations within a single analytic framework. Second, although engagement is widely understood to include behavioral, emotional, and cognitive dimensions, it is still frequently analyzed as a unitary construct, which may obscure the distinct roles of its dimensions (5, 6). Third, most available evidence emphasizes average associations, with less attention to questions of greater practical relevance, such as which learning environmental domains matter most after adjustment for student background, the extent to which engagement statistically accounts for the cross-sectional association between perceived learning environment and learning gains, and whether substantial student subgroups remain poorly served despite overall positive patterns (4). Fourth, much of the literature is based on single-wave cross-sectional data, making it difficult to determine whether observed associations reflect stable institutional patterns or cohort-specific circumstances.

Accordingly, this study drew on four consecutive years (2021–2024) of National College Student Survey data from a medical university in western China (*N* = 11,717) to examine associations among perceived learning environment, student engagement, and learning gains. The National College Student Survey (NCSS) afforded continuous, multidimensional, and structured measures of students’ learning experiences across four annual survey waves, enabling cross-cohort analyses of these relationships within a single institutional context. Guided by the I-E-O framework, the concept of proximal processes, and self-determination theory, we modeled perceived learning environment across five domains, operationalized student engagement as 3 distinct dimensions of behavioral, emotional, and cognitive, and specified learning gains as the focal educational outcome. We further aimed to quantify the incremental explanatory contribution of each learning environment domain to each engagement dimension beyond student background characteristics, examine the extent to which behavioral, emotional, and cognitive engagement statistically account for the association between the perceived learning environment and learning gains, and identify, through person-centered cluster analysis, student profiles characterized by distinct configurations of environment perceptions, engagement, and learning gains. In addition, year-stratified sensitivity analyses were conducted to assess the stability of the observed associations across survey waves.

## 2 Methods

### 2.1 Study design, data source, and analytic sample

We conducted a pooled repeated cross-sectional study using data from 4 consecutive administrations (2021–2024) of the NCSS at a medical university in western China. The NCSS was administered online and anonymously to undergraduate students across Years 1–5 and academic programs, and participation was voluntary. Each survey record represented a student’s reported educational experience in the corresponding survey year. Because the survey was anonymous and contained no stable identifier, responses could not be linked across survey waves.

The de-identified dataset available for the present study contained 11,728 survey records: 1,015 from 2021, 4,227 from 2022, 3,028 from 2023, and 3,458 from 2024. Eleven student responses coded as “graduated/other” on the year-of-study item were excluded (0, 5, 0, and 6 by wave), yielding a final analytic sample of 11,717 student responses (Figure 1; 1,015, 4,222, 3,028, and 3,452, respectively). The numbers of eligible or invited students for each survey wave were not consistently retained in the archived records; therefore, formal annual response rates could not be calculated.

**Figure 1:**
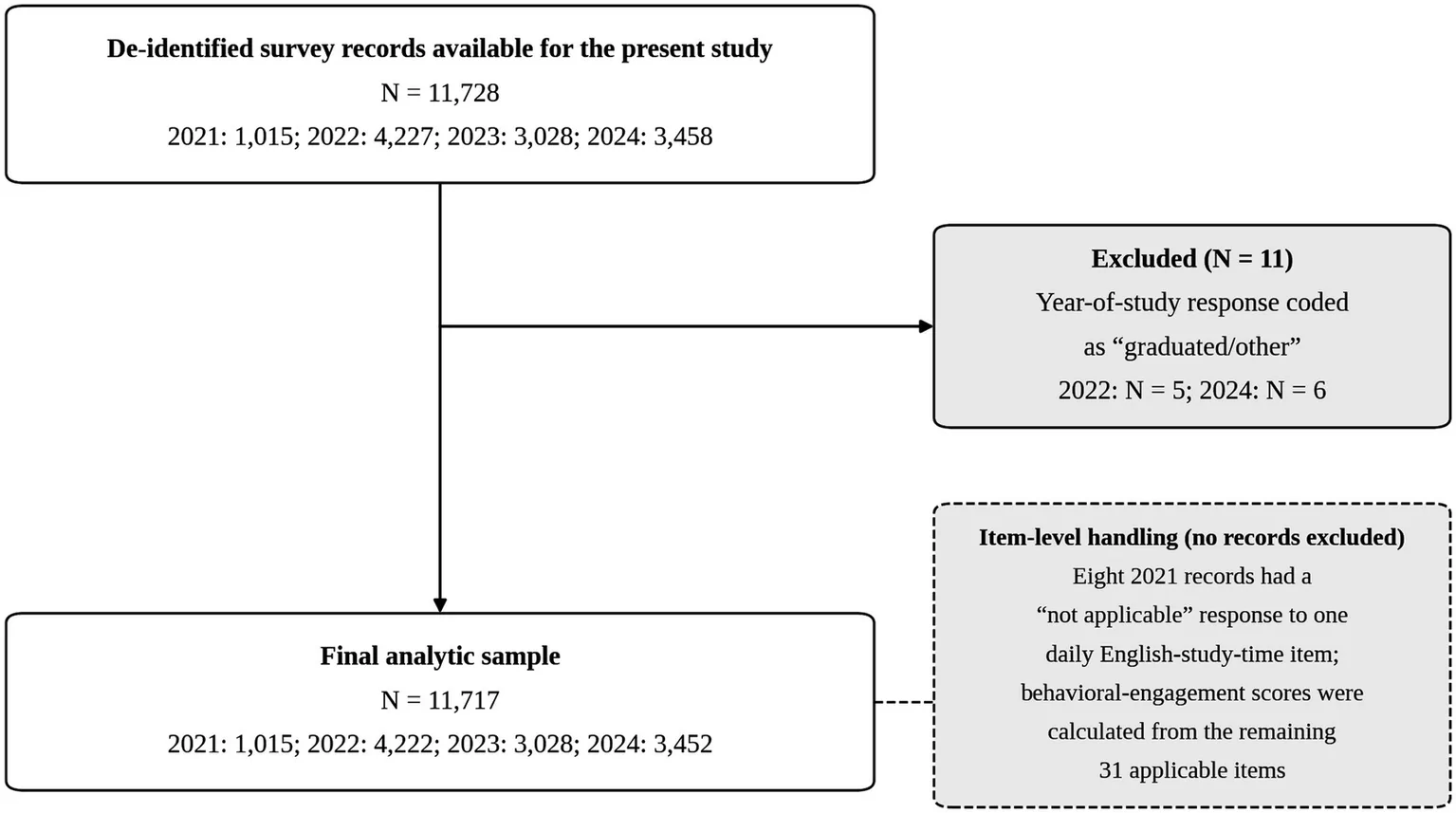
Flow of record selection for the analytic sample.

To assess the comparability of the four survey waves before pooling, we examined between-year differences in the core study variables and cohort composition (Supplementary Table S1). Between-year differences in the core study variables were negligible (*η*^2^ = 0.0019–0.0077). Differences in cohort composition were generally modest, with the largest difference observed for program type (Cramér’s *V* = 0.165), which was included as a covariate in all models. The four survey waves were therefore pooled for the primary analyses, with survey year included as an adjustment covariate in pooled regression-based models. Year-stratified analyses were additionally conducted to examine the consistency of the observed associations across survey waves.

### 2.2 Measures

All focal constructs were derived from the NCSS, a Chinese undergraduate learning survey used to assess students’ learning experiences, engagement, and developmental outcomes. Unless otherwise specified, items used 6-point response options, and scale scores were computed as the mean of constituent items, with higher scores indicating more favorable perceptions, higher engagement, or greater learning gains. Internal consistency (Cronbach’s *α*) and descriptive statistics for each subscale are reported in Supplementary Table S2 (*α* = 0.72–0.93).

To reduce potential common method effects, the survey was administered anonymously, and the focal constructs were assessed using distinct item content and response formats, including satisfaction, frequency, agreement, and perceived-gain ratings. Common method variance was assessed using Harman’s single-factor test, with all 141 items entered into an unrotated principal-component analysis.

#### 2.2.1 Learning environment

Students’ perceptions of the learning environment were assessed using 48 items covering 5 domains: classroom teaching quality, facilities and resources, developmental support, interpersonal support, and teaching administration. These items evaluated students’ satisfaction with relevant aspects of the teaching and learning environment on a 6-point scale ranging from 1 = very dissatisfied to 6 = very satisfied. Classroom teaching quality primarily reflected teachers’ commitment to teaching, classroom organization, and approaches to course assessment. Facilities and resources referred to teaching and practical training facilities, as well as learning and living spaces. Developmental support included academic guidance, career planning, and developmental opportunities. Interpersonal support focused on teacher–student relationships, peer support, and the social climate within the class or grade. Teaching administration reflected teaching arrangements, information communication, and management systems. Internal consistency was good across the 5 domains, with Cronbach’s α ranging from 0.84 to 0.93. Domain scores were calculated as the mean of the corresponding items (Supplementary Table S2).

#### 2.2.2 Student engagement

Student engagement was measured across three dimensions: behavioral engagement, emotional engagement, and cognitive engagement. Behavioral engagement comprised 32 items covering classroom participation, extracurricular practice, reading and writing activities, peer learning, and learning time. Study-time items assessed average daily time using six ordered categories ranging from <1 h to >12 h; the remaining items used a 6-point frequency scale ranging from 1 = never to 6 = always. Emotional engagement included 13 items reflecting students’ interest in their major and learning, perceived value, and emotional attachment. Cognitive engagement included 19 items capturing deep thinking, self-monitoring, and the use of learning strategies. Both emotional and cognitive engagement were measured using a 6-point agreement scale ranging from 1 = strongly disagree to 6 = strongly agree. Ten negatively worded engagement items were reverse-scored before scale scores were calculated. In the 2021 questionnaire, one item assessing daily English-study time included the response option “I am a foreign-language major; not applicable”; eight respondents selected this option. These responses were treated as item-level missing, and behavioral engagement scores for these respondents were calculated from the remaining 31 items; no imputation was performed. Scores for each engagement dimension were calculated as item means. Overall engagement was calculated as the mean of the scores on three dimensions. Internal consistency was good across the three dimensions (Cronbach’s *α* = 0.84–0.90). The dimensions were positively intercorrelated (*r* = 0.294–0.599; all *p* < 0.001), supporting their treatment as related but distinct constructs (4).

#### 2.2.3 Learning gains

Learning gains were assessed using 29 NCSS-derived items across six domains: professional competence, values and character, interest development, learning ability, communication and collaboration, and overall self-evaluation. Items were rated on a 6-point perceived-gain scale ranging from 1 = no gain to 6 = very substantial gain. Internal consistency was acceptable to excellent across the 6 domains, with Cronbach’s α ranging from 0.72 to 0.93. Domain scores were calculated as item means, with higher scores indicating greater self-perceived learning and developmental gains. For the cross-sectional indirect association analysis, a composite learning-gain score was calculated as the mean of the 6 domain scores.

#### 2.2.4 Covariates

Prespecified covariates included survey year, sex (male or female), year of study (Years 1–5), ethnicity (Han or minority), rural/urban origin (urban or rural), parental educational attainment (high school or below vs. junior college or above), household income, and program type. Household income was classified as low (≤6,000 RMB/month), middle (6,001–14,000 RMB/month), or high (>14,000 RMB/month). Programs were grouped according to training objectives, curriculum structure, and practical-training requirements into clinical medical, non-clinical medical and health-related, and non-medical programs; the classification of individual programs is provided in the Supplementary Table S8.

Categorical covariates were dummy coded, with 2021, male sex, Year 1, Han ethnicity, urban origin, high school or lower parental education, low household income, and non-medical program as the reference categories. The online survey required a response to each item before submission. Apart from the eight item-level “not applicable” responses described above, all focal scale scores and covariates were complete in the analytic sample of 11,717 student responses, and no respondent-level imputation was required.

### 2.3 Statistical analysis

Analyses were conducted using R, version 4.4.3 (R Foundation for Statistical Computing, Vienna, Austria), and IBM SPSS Statistics, version 28.0 (IBM Corp, Armonk, New York), using two-sided tests with α = 0.05.

#### 2.3.1 Descriptive statistics and group comparisons

We summarized continuous variables using mean (*SD*). Between-group differences in learning environment, engagement dimensions, and learning gains were examined using Welch’s *t*-tests or one-way ANOVA as appropriate. We reported Cohen’s *d* for two-group comparisons and *η*^2^ for ANOVA.

#### 2.3.2 Hierarchical multiple linear regression

To quantify the incremental contribution of the learning environment beyond background characteristics, we fitted hierarchical multiple linear regression models predicting each engagement dimension (behavioral, emotional, cognitive). Model 1 included survey year and all covariates; Model 2 added the five learning environment domains. Categorical predictors were entered using dummy coding with prespecified reference categories. We reported standardized coefficients (*β*) with 95% confidence intervals, *p*-values, *R*^2^, and Δ*R*^2^. Multicollinearity was assessed using variance inflation factors, with a prespecified threshold of less than 5.

#### 2.3.3 Cross-sectional indirect association analysis

We fitted a parallel path model in which behavioral, emotional, and cognitive engagement were entered simultaneously as intermediate variables in the cross-sectional association between overall learning environment and overall learning gains, with the same prespecified covariates included in all model equations. Continuous focal variables were *z*-standardized, whereas dummy-coded covariates retained their original coding. Specific indirect associations, the total indirect association, the direct association, and the total association were estimated with percentile bootstrap 95% confidence intervals based on 10,000 resamples. The proportion of the total association accounted for by the total indirect association was also calculated.

#### 2.3.4 Person-centered profiling using k-means clustering

To characterize heterogeneity in the joint distribution of learning environment, engagement, and learning gains, we conducted k-means clustering on five *z*-standardized indicators: overall learning environment, behavioral engagement, emotional engagement, cognitive engagement, and overall learning gains. A three-profile solution was fitted using 50 random starts, a maximum of 100 iterations, and a fixed seed of 12345. Cluster separation was descriptively assessed using the mean silhouette coefficient calculated in a fixed random sample of 2,000 records. Profiles were ordered according to the mean of their five standardized centroids. Background characteristics across profiles were compared using Pearson *χ*^2^ tests, with Cramér’s *V* reported as the effect-size index.

#### 2.3.5 Interaction and year-stratified analyses

To examine whether the principal associations differed across selected student characteristics, we conducted six prespecified interaction tests: classroom teaching quality × year of study for each of the three engagement dimensions, interpersonal support × program type for emotional engagement, developmental support × parental education for cognitive engagement, and overall learning environment × sex for overall engagement. Each interaction was evaluated using an omnibus *F* test. Statistical significance was assessed at the nominal *α* = 0.05 level and, for the six interaction tests, at a Bonferroni-adjusted threshold of *α* = 0.0083. To examine cross-wave consistency, the full regression models were refitted separately within each survey wave using the same variable definitions and covariates applicable within that wave. HC3 heteroskedasticity-consistent standard errors were additionally examined as a sensitivity analysis.

## 3 Results

### 3.1 Sample characteristics and scale descriptives

The analytic sample comprised 11,717 student responses. Most respondents were female (7,990; 68.2%), 3,770 (32.2%) were from ethnic minority groups, and first- through fifth-year students accounted for 32.2%, 28.5%, 28.3%, 9.0%, and 2.0% of the sample, respectively. By program type, 5,542 student responses (47.3%) were from clinical medical programs, including clinical medicine, pediatrics, stomatology, anesthesiology, and medical imaging; 4,867 (41.5%) were from non-clinical medical and health-related programs, including nursing, pharmacy, preventive medicine, clinical pharmacy, and rehabilitation; and 1,308 (11.2%) were from non-medical programs, including public administration, information management, social work, and English (Table 1). Thus, the analytic sample included undergraduates from both medical/health-related and non-medical programs within a medical university.

**Table 1:** Participant characteristics of undergraduate students at a medical university in western China, 2021–2024 (*N* = 11,717).

Characteristic	Category	*N*	%
Sex	Male	3,727	31.8
	Female	7,990	68.2
Ethnicity	Han	7,947	67.8
	Ethnic minority	3,770	32.2
Year of study	Year 1	3,777	32.2
	Year 2	3,336	28.5
	Year 3	3,315	28.3
	Year 4	1,052	9.0
	Year 5	237	2.0
Program type	Clinical medical	5,542	47.3
	Non-clinical medical and health-related	4,867	41.5
	Non-medical	1,308	11.2
Rural/urban origin	Urban	3,497	29.8
	Rural	8,220	70.2
Highest parental education	High school or below	9,296	79.3
	Junior college or above	2,421	20.7
Household income	Low	7,747	66.1
	Middle	3,238	27.6
	High	732	6.2

Percentages may not sum to 100 due to rounding.

Consistent with the regional context, most participants were of rural origin (8,220; 70.2%) rather than urban areas (29.8%). Parental educational attainment was modest: 79.3% reported a highest parental education level of high school or below, and 20.7% reported a level of junior college or above. Self-reported household income was concentrated in the lower brackets: 66.1% reported low income, 27.6% middle, and 6.2% high. Collectively, these distributions characterize a university sample marked by substantial rural representation and comparatively limited family educational and economic capital (Table 1). In Harman’s single-factor test, the first unrotated component accounted for 30.4% of the total variance (*N* = 11,709 responses with complete item data), below the conventional 50% criterion.

### 3.2 Differences across student background characteristics

Differences in the three composite scores across background characteristics were small (Supplementary Table S3). For overall engagement, the largest contrasts were observed by parental education (junior college or above vs. high school or below: 3.91 vs. 3.85; |*d*| = 0.131), sex (female vs. male: 3.88 vs. 3.83; |*d*| = 0.110), and household income (low, middle, and high: 3.84, 3.89, and 4.02; *η*^2^ = 0.0095). Household income showed a similar gradient for learning gains (4.39, 4.47 and 4.60; *η*^2^ = 0.0070). Students in non-medical programs also reported slightly lower learning gains than those in clinical medical and non-clinical medical and health-related programs (*η*^2^ = 0.0033). Learning environment did not differ by sex, ethnicity, rural/urban origin, or parental education (all |*d*| ≤ 0.024; all *p* ≥ 0.32) and showed only small differences by year of study, household income, and program type (*η*^2^ ≤ 0.0052). Survey year accounted for less than 0.6% of the variance in each composite score (*η*^2^ = 0.0020–0.0055).

All observed effect sizes were negligible to small (|*d*| < 0.20; *η*^2^ < 0.0100). Background characteristics were therefore associated with statistically detectable but limited differences in students’ reported learning experiences.

### 3.3 Associations of learning environment with student engagement

Survey year and background characteristics explained 4.0%, 2.9%, and 1.5% of the variance in behavioral, emotional, and cognitive engagement, respectively. Adding the five learning environment domains increased the explained variance by 22.5%, 27.6%, and 23.1% (all *p* < 0.001), yielding full-model *R*^2^ values of 0.265, 0.305, and 0.246 (Table 2). All variance inflation factors were below 5 (maximum 4.75), indicating no substantial multicollinearity. The five learning environment domains thus accounted for considerably more variance in engagement than the measured background characteristics.

**Table 2:** Hierarchical multiple linear regression predicting student engagement from learning environment among undergraduate students at a medical university in western China, 2021–2024.

Predictor	Behavioral engagement	Emotional engagement	Cognitive engagement
Covariates			
Survey year (2022 vs. 2021)	0.052 (−0.007, 0.112)	−0.143^***^ (−0.201, −0.085)	−0.096^**^ (−0.156, −0.035)
Survey year (2023 vs. 2021)	0.060 (−0.002, 0.121)	−0.272^***^ (−0.331, −0.212)	−0.131^***^ (−0.194, −0.069)
Survey year (2024 vs. 2021)	0.116^***^ (0.055, 0.177)	−0.297^***^ (−0.356, −0.238)	−0.080^*^ (−0.142, −0.018)
Sex (female vs. male)	−0.048^**^ (−0.082, −0.014)	0.244^***^ (0.211, 0.278)	0.020 (−0.015, 0.054)
Year of study (Year 2 vs. Year 1)	0.197^***^ (0.157, 0.238)	0.038 (−0.001, 0.078)	0.043^*^ (0.002, 0.084)
Year of study (Year 3 vs. Year 1)	0.336^***^ (0.295, 0.376)	0.024 (−0.015, 0.064)	0.114^***^ (0.073, 0.155)
Year of study (Year 4 vs. Year 1)	0.433^***^ (0.373, 0.494)	0.004 (−0.055, 0.063)	0.150^***^ (0.089, 0.211)
Year of study (Year 5 vs. Year 1)	0.356^***^ (0.242, 0.470)	0.130^*^ (0.019, 0.241)	0.181^**^ (0.065, 0.296)
Ethnicity (ethnic minority vs. Han)	−0.024 (−0.057, 0.010)	−0.056^***^ (−0.088, −0.023)	−0.059^***^ (−0.093, −0.026)
Rural/urban origin (rural vs. urban)	−0.046^*^ (−0.084, −0.007)	−0.002 (−0.040, 0.035)	−0.052^**^ (−0.091, −0.013)
Parental education (junior college or above vs. high school or below)	0.146^***^ (0.102, 0.189)	−0.049^*^ (−0.091, −0.006)	0.006 (−0.038, 0.050)
Household income (middle vs. low)	0.036 (−0.001, 0.073)	0.020 (−0.016, 0.056)	0.058^**^ (0.020, 0.095)
Household income (high vs. low)	0.319^***^ (0.250, 0.388)	0.025 (−0.042, 0.093)	0.269^***^ (0.199, 0.339)
Program type (clinical medical vs. non-medical)	−0.068^*^ (−0.122, −0.014)	0.058^*^ (0.005, 0.110)	−0.040 (−0.094, 0.015)
Program type (non-clinical medical and health-related vs. non-medical)	−0.042 (−0.095, 0.011)	−0.003 (−0.054, 0.049)	−0.051 (−0.104, 0.003)
Learning environment			
Classroom teaching quality	0.350^***^ (0.324, 0.377)	0.351^***^ (0.325, 0.377)	0.287^***^ (0.260, 0.314)
Facilities and resources	0.010 (−0.019, 0.039)	−0.118^***^ (−0.146, −0.090)	0.012 (−0.017, 0.042)
Developmental support	0.236^***^ (0.202, 0.270)	0.160^***^ (0.127, 0.193)	0.175^***^ (0.141, 0.210)
Interpersonal support	0.008 (−0.019, 0.035)	0.121^***^ (0.095, 0.147)	0.079^***^ (0.051, 0.106)
Teaching administration	−0.128^***^ (−0.158, −0.098)	0.042^**^ (0.013, 0.071)	−0.035^*^ (−0.065, −0.005)
Model fit			
*R*^2^ (Model 1)	0.040	0.029	0.015
*R*^2^ (Model 2)	0.265	0.305	0.246
Δ*R*^2^	0.225	0.276	0.231
*p* for Δ*R*^2^	<0.001	<0.001	<0.001

Coefficients are standardized (β); 95% confidence intervals are shown in parentheses below the coefficients. For categorical covariates, the category after “vs” is the reference. Δ*R*^2^ represents the incremental variance explained by adding learning environment domains (Model 2) to the covariate model (Model 1). ^*^p < 0.05, ^**^p < 0.01, ^***^p < 0.001.

Classroom teaching quality had the largest positive coefficient for each engagement dimension (behavioral: *β* = 0.350, 95% CI 0.324–0.377; emotional: *β* = 0.351, 95% CI 0.325–0.377; cognitive: *β* = 0.287, 95% CI 0.260–0.314), followed by developmental support (*β* = 0.236, 0.160, and 0.175, respectively; all *p* < 0.001). Interpersonal support was positively associated with emotional (*β* = 0.121, 95% CI 0.095–0.147) and cognitive engagement (*β* = 0.079, 95% CI 0.051–0.106) but not with behavioral engagement (*β* = 0.008, 95% CI − 0.019 to 0.035). With the other domains held constant, facilities and resources were negatively associated with emotional engagement (*β* = −0.118, 95% CI −0.146 to −0.090), and teaching administration was negatively associated with behavioral (*β* = −0.128, 95% CI −0.158 to −0.098) and cognitive engagement (*β* = −0.035, 95% CI −0.065 to −0.005) but positively associated with emotional engagement (*β* = 0.042, 95% CI 0.013–0.071).

### 3.4 Cross-sectional indirect associations between learning environment and learning gains through student engagement

In the cross-sectional parallel decomposition model, the total association between learning environment and learning gains was 0.734 (95% bootstrap CI 0.719–0.749), and the direct association was 0.500 (0.480–0.519). The total indirect association through the three engagement dimensions was 0.234 (0.221–0.248), corresponding to 31.93% of the total association (Table 3 and Figure 2). Each specific indirect association was positive, with a confidence interval excluding zero: 0.073 through behavioral, 0.089 through emotional, and 0.072 through cognitive engagement. The indirect association through emotional engagement exceeded those through behavioral (difference 0.016; 95% CI 0.004–0.027) and cognitive engagement (difference 0.017; 95% CI 0.002–0.032), although these differences were small in absolute terms. In the domain-specific models, emotional engagement showed the largest specific indirect association for every learning environment domain (Supplementary Table S4). Across the six learning-gain domains, all specific indirect associations were positive; however, the confidence interval included zero for the path through emotional engagement to overall self-evaluation (0.006; 95% CI − 0.003 to 0.015; Supplementary Table S5). Because all variables were measured at the same time point, these estimates decompose contemporaneous associations and do not establish temporal mediation.

**Table 3:** Decomposition of the cross-sectional association between learning environment and learning gains through student engagement, 2021–2024.

Component	Estimate	Bootstrap *SE*	95% CI	*p*-value	Proportion of total association, %
Behavioral engagement	0.073	0.004	0.065, 0.082	<0.001	9.99
Emotional engagement	0.089	0.005	0.080, 0.098	<0.001	12.14
Cognitive engagement	0.072	0.005	0.063, 0.081	<0.001	9.80
Total indirect association	0.234	0.007	0.221, 0.248	<0.001	31.93
Direct association	0.500	0.010	0.480, 0.519	<0.001	–
Total association	0.734	0.008	0.719, 0.749	<0.001	–

Estimates represent adjusted cross-sectional associations. Models were adjusted for survey year, sex, year of study, ethnicity, rural/urban origin, parental education, household income, and program type. Percentile bootstrap 95% CIs were based on 10,000 resamples. Overall learning environment and learning gains were calculated as equal-weighted means of their respective domain scores.

**Figure 2:**
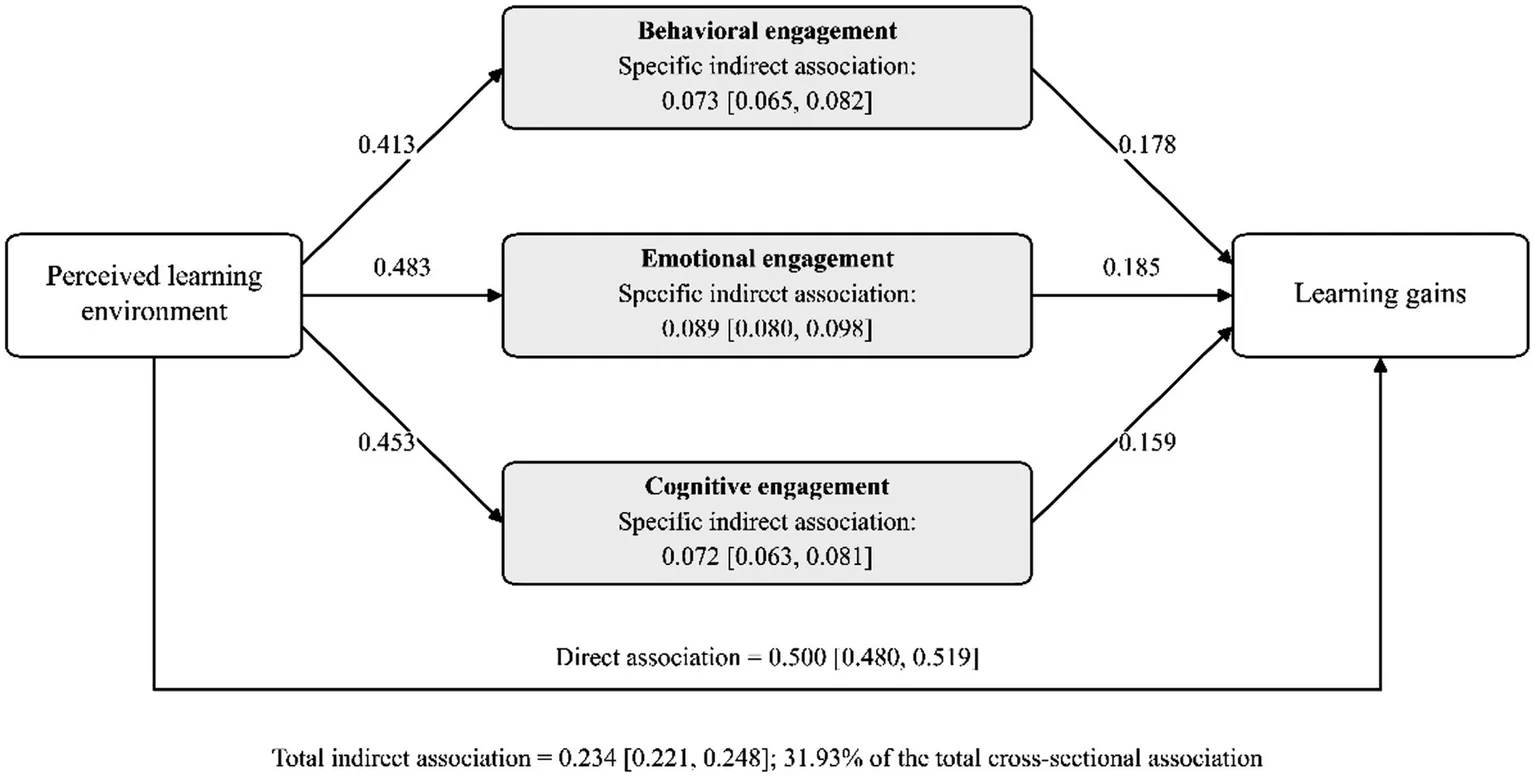
Cross-sectional indirect association model. Values along the arrows are standardized regression coefficients, and values within the engagement boxes are specific indirect associations, and brackets show 95% percentile-bootstrap confidence intervals based on 10,000 resamples. Paths from each engagement dimension to learning gains are adjusted for the learning environment and the other two engagement dimensions, and all paths are adjusted for survey year, sex, year of study, ethnicity, rural/urban origin, parental education, household income, and program type. Arrows represent regression paths specified for statistical decomposition and do not imply temporal or causal ordering.

### 3.5 Assessment of effect modification across student subgroups

To probe whether associations between learning environment and student engagement varied across subgroups, we fitted interaction models testing year of study, program type, parental education, and sex as potential moderators. None of the six interaction terms was statistically significant (nominal *p* = 0.082–0.687; all Bonferroni-adjusted *p* ≥ 0.49; Supplementary Table S6). We found no evidence that the associations of classroom teaching quality with the three engagement dimensions differed by year of study, or that the associations of interpersonal support differed by program type, developmental support by parental education, or the overall learning environment by sex. Overall, the tested environment–engagement associations were broadly similar across these student subgroups.

### 3.6 Sensitivity analysis: cross-year stability of associations

The four survey waves showed only negligible between-year differences in the core study variables (all *η*^2^ ≤ 0.0077), with broadly similar cohort composition overall (Supplementary Table S1). Year-stratified models reproduced the pooled pattern (Table 4). Within each wave, background characteristics explained 1.3–5.7% of the variance in engagement, whereas the five environment domains added 15.5–31.4% (all *p* < 0.001). Classroom teaching quality was positively and significantly associated with every engagement dimension in every wave (12 of 12 models; *β* = 0.210–0.420). Developmental support was positive in all 12 models and statistically significant in 11 (10 when heteroskedasticity-consistent HC3 standard errors were used; Supplementary Table S7); the exception was cognitive engagement in 2021 (*β* = 0.049, *p* = 0.396). Coefficients for the other three domains were smaller and less consistent across waves (Supplementary Table S7). These results indicate that the principal associations were not attributable to any single survey year.

**Table 4:** Year-stratified hierarchical regression model fit for engagement outcomes.

Outcome	Year	*N*	*R*^2^ (Model 1)	*R*^2^ (Model 2)	Δ*R*^2^	*p* for Δ*R*^2^
Behavioral engagement	2021	1,015	0.057	0.212	0.155	<0.001
	2022	4,222	0.034	0.263	0.229	<0.001
	2023	3,028	0.032	0.260	0.229	<0.001
	2024	3,452	0.049	0.288	0.239	<0.001
Emotional engagement	2021	1,015	0.014	0.323	0.308	<0.001
	2022	4,222	0.034	0.291	0.257	<0.001
	2023	3,028	0.041	0.301	0.259	<0.001
	2024	3,452	0.014	0.328	0.314	<0.001
Cognitive engagement	2021	1,015	0.022	0.216	0.194	<0.001
	2022	4,222	0.013	0.236	0.224	<0.001
	2023	3,028	0.021	0.237	0.216	<0.001
	2024	3,452	0.015	0.283	0.268	<0.001

Models were fitted separately within each survey wave. Model 1 included background covariates (sex, year of study, ethnicity, rural/urban origin, parental education, household income, and program type); Model 2 added the five learning environment domains. *R*^2^, proportion of variance in the outcome explained by the model; Δ*R*^2^, increase in *R*^2^ from Model 1 to Model 2; *p* for Δ*R*^2^, from the *F* test for the change in *R*^2^. *N*, number of responses in each survey wave.

### 3.7 Student profiles derived from person-centered analyses

The three-profile k-means solution had a mean silhouette coefficient of 0.225 (Table 5 and Figure 3). Profile 1 (*N* = 4,360, 37.2%) scored below the sample mean on all five indicators (*z* = −0.84 to −0.60) and was characterized as the convergent-low profile. Profile 2 (*N* = 5,067, 43.2%) scored close to the sample mean (*z* = 0.04–0.20) and was characterized as the intermediate profile, whereas Profile 3 (*N* = 2,290, 19.5%) scored above the mean on all indicators (*z* = 1.05–1.27) and was characterized as the convergent-high profile. The profiles thus formed an ordered gradient in which learning environment, engagement, and learning gains varied together.

**Table 5:** Mean (SD) of core study variables by student profile.

Variable	Profile 1: convergent-low (*N* = 4,360)	Profile 2: intermediate (*N* = 5,067)	Profile 3: convergent-high (*N* = 2,290)	*F*
Learning environment	3.98 (0.46)	4.59 (0.42)	5.19 (0.50)	5,640.85^***^
Behavioral engagement	2.85 (0.48)	3.23 (0.43)	3.82 (0.57)	3,157.31^***^
Emotional engagement	4.00 (0.45)	4.62 (0.44)	5.15 (0.48)	5,233.63^***^
Cognitive engagement	3.51 (0.37)	3.94 (0.32)	4.57 (0.45)	6,335.05^***^
Learning gains	3.86 (0.46)	4.53 (0.39)	5.28 (0.46)	8,345.90^***^

Values are mean (SD) on the original 1–6 scale. F statistics are from one-way ANOVA and are descriptive, because the profiles were derived from these variables. ***p < 0.001.

**Figure 3:**
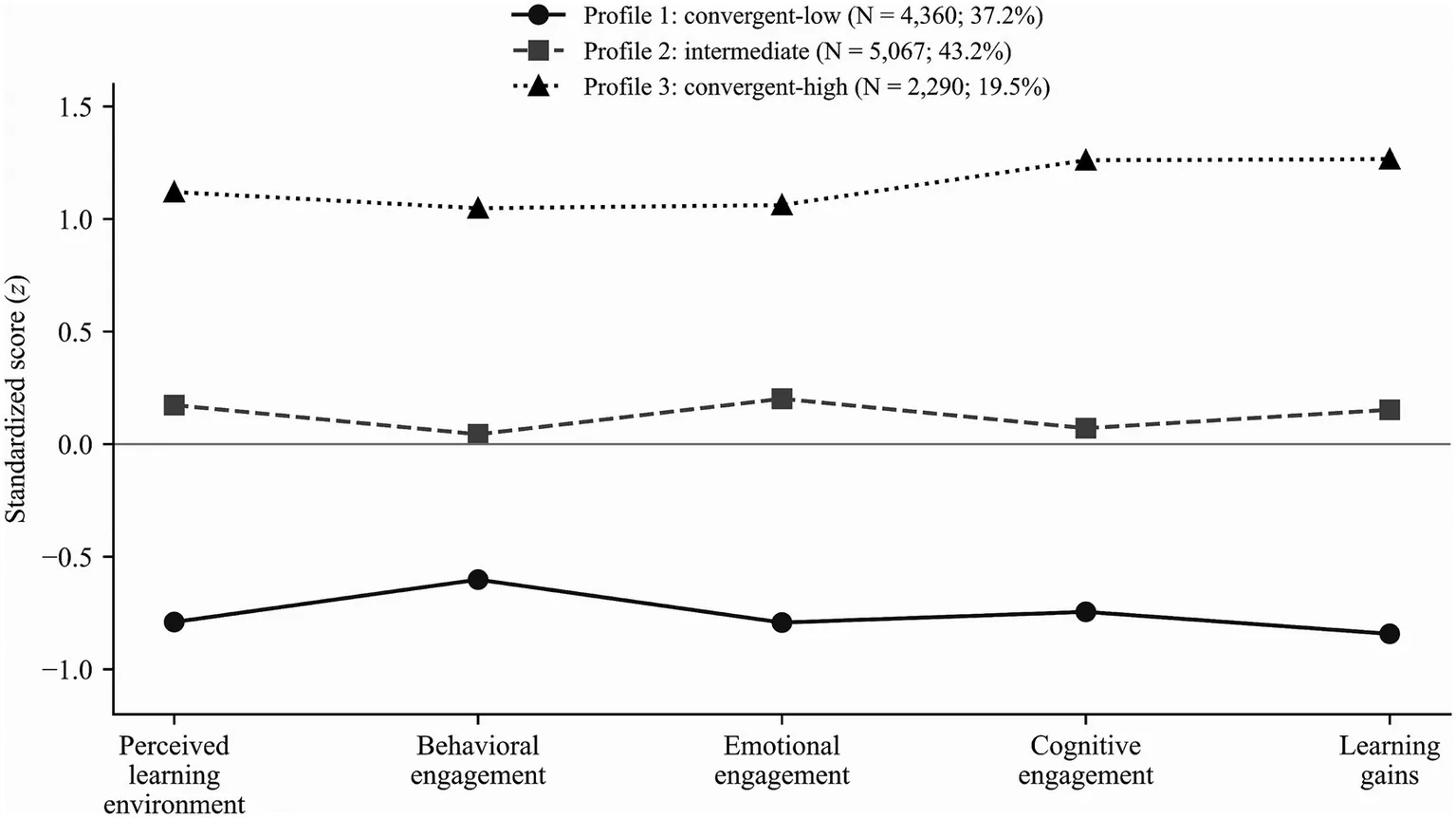
Student profiles across standardized learning environment, engagement, and learning-gain indicators. Notes: Profiles were derived by k-means clustering of five z-standardized indicators: overall learning environment, behavioral engagement, emotional engagement, cognitive engagement, and overall learning gains. Values are *z* scores relative to the full sample (*N* = 11,717). Lines connect profile means across indicators for visualization only.

Profile membership was associated with sex, rural/urban origin, parental education, household income, and program type (all *p* < 0.001), but all associations were weak (Cramér’s *V* = 0.033–0.063; Table 6). Compared with Profile 3, Profile 1 included higher proportions of rural-origin students (70.1% vs. 65.0%) and low-income students (69.2% vs. 60.1%), and a lower proportion of students whose parents had junior college education or above (20.2% vs. 24.7%). Ethnicity was not associated with profile membership (*p* = 0.055). Thus, although profile membership differed statistically across several background characteristics, these characteristics only weakly differentiated the three profiles. The program classification used in these analyses is detailed in Supplementary Table S8.

**Table 6:** Distribution of selected background characteristics across student profiles among undergraduate students at a medical university in western China, 2021–2024 (*N* = 11,717).

Characteristic	Profile 1: convergent-low (*N* = 4,360)	Profile 2: intermediate (*N* = 5,067)	Profile 3: convergent-high (*N* = 2,290)	*χ* ^2^	*p*	Cramér’s *V*
Female	2,850 (65.4)	3,590 (70.9)	1,550 (67.7)	32.82	<0.001	0.053
Ethnic minority	1,448 (33.2)	1,628 (32.1)	694 (30.3)	5.82	0.055	0.022
Rural origin	3,056 (70.1)	3,676 (72.5)	1,488 (65.0)	43.18	<0.001	0.061
Parental education: junior college or above	882 (20.2)	974 (19.2)	565 (24.7)	29.37	<0.001	0.050
Household income				93.69	<0.001	0.063
Low	3,016 (69.2)	3,355 (66.2)	1,376 (60.1)			
Middle	1,115 (25.6)	1,437 (28.4)	686 (30.0)			
High	229 (5.3)	275 (5.4)	228 (10.0)			
Program type				24.82	<0.001	0.033
Non-medical	564 (12.9)	530 (10.5)	214 (9.3)			
Non-clinical medical and health-related	1,796 (41.2)	2,113 (41.7)	958 (41.8)			
Clinical medical	2,000 (45.9)	2,424 (47.8)	1,118 (48.8)			

Values are presented as *N* (%). For binary variables, values represent the count and percentage in the named category. Pearson *χ*^2^ tests.

## 4 Discussion

This study had three main findings. First, after accounting for survey year and background characteristics, the five learning-environment domains explained a substantial additional proportion of the variance in engagement; classroom teaching quality showed the largest associations with all three engagement dimensions in the pooled analysis and was significantly associated with each dimension in every survey wave, while developmental support also showed consistently positive associations. Second, behavioral, emotional, and cognitive engagement together statistically accounted for about one-third of the cross-sectional association between learning environment and learning gains, with engagement dimensions showing distinct patterns of association across learning-gain domains. Third, the main environment–engagement associations were broadly consistent across survey waves, but more than one-third of the analytic sample belonged to a profile with concurrently low scores on learning environment, engagement, and learning gains, and background characteristics only weakly differentiated this profile.

### 4.1 Learning environment domains most closely associated with engagement

Survey year and background characteristics explained only 1.5–4.0% of the variance in engagement, whereas adding the five learning-environment domains explained an additional 22.5–27.6%. This pattern is consistent with the input–environment–outcome framework, which holds that student development is related not only to the characteristics students bring on entry but also to the educational environment they experience in college (7), and with previous research in health professions education showing that students’ perceptions of the learning environment are closely associated with their engagement (13). The present findings further show that this association was not distributed equally across domains: classroom teaching quality showed the largest associations with all three engagement dimensions, developmental support was consistently and positively associated with all three, interpersonal support was associated mainly with emotional and cognitive engagement, and facilities and resources and teaching administration showed null, negative, or mixed associations after adjustment for the other domains. An overall environment score may therefore summarize students’ general appraisal of their educational environment, but identifying specific targets for quality improvement requires distinguishing among learning environment domains.

Classroom teaching quality and developmental support were the two domains most consistently associated with engagement, and both were positively associated with all three engagement dimensions in every survey wave. These two domains more directly reflect the educational processes that students experience. Classroom teaching quality captures teachers’ commitment to teaching, classroom organization, and approaches to course assessment, whereas developmental support captures academic guidance, career planning, and opportunities for academic and personal development. This pattern is consistent with Bronfenbrenner and Morris’s concept of proximal processes, which holds that development occurs primarily through sustained and recurring interactions between learners and their immediate environment (8). Research in medical education has likewise shown that formative feedback during teaching and ongoing learning support are closely associated with student engagement (14, 15).

By contrast, the associations of the other three domains with engagement were more limited or selective. Interpersonal support was positively associated with emotional and cognitive engagement but showed no independent association with behavioral engagement after adjustment for the other domains. This selective pattern may reflect what the domain measured: interpersonal support captured a broader relational environment, including teacher–student relationships, peer support, and class climate, whereas instructional feedback in class was captured by the classroom teaching quality domain. In models including both domains, the coefficient for interpersonal support therefore represents the component of relational experience not shared with classroom teaching quality, and this component showed stronger associations with students’ emotional connection to learning and deeper cognitive engagement than with behavioral participation in learning (11, 16). After adjustment for the other domains, facilities and resources were negatively associated only with emotional engagement and were not significantly associated with behavioral or cognitive engagement, whereas teaching administration was negatively associated with behavioral and cognitive engagement and weakly positively associated with emotional engagement. Given the intercorrelations among the learning environment domains, these coefficients should be interpreted as conditional associations within the multidomain model. The more consistent finding was that neither facilities and resources nor teaching administration showed an additional positive association with engagement across dimensions once domains more closely embedded in the learning process were taken into account.

These domain-specific patterns point to different priorities for educational improvement. For classroom teaching quality, improvement could focus on classroom organization and approaches to course assessment, particularly on ensuring that assessment and feedback serve the learning process (15). For developmental support, academic guidance, career planning, and developmental opportunities could be integrated more closely with students’ regular learning (11). For interpersonal support, initiatives to improve teacher–student relationships, peer support, and class climate may be particularly relevant to students’ emotional and cognitive engagement (16). For facilities and resources and teaching administration, once basic provision is in place, improvement may focus on how existing resources and administrative arrangements can better support students’ learning.

### 4.2 Engagement as a partial and multidimensional link between learning environment and learning gains

The second main finding was that behavioral, emotional, and cognitive engagement together statistically accounted for 31.93% of the cross-sectional association between learning environment and learning gains. Previous research in health professions education has shown that engagement is closely associated with both the educational environment and academic performance and may account for part of the association between them (13). The present finding is also consistent with engagement research and self-determination theory, according to which supportive aspects of the learning environments are associated with greater student engagement, which is in turn associated with deeper learning and development (4, 11). At the same time, the direct association remained considerably larger than the total indirect association, indicating that the three measured engagement dimensions accounted for only part of the cross-sectional association between learning environment and learning gains.

The pattern of indirect associations was also multidimensional. In the overall model, the three specific indirect associations were similar in magnitude; the specific indirect association through emotional engagement was the largest, but differed only modestly from those through behavioral and cognitive engagement. When learning gains were examined by domain, the relative pattern changed (Supplementary Table S5): the indirect association through behavioral engagement was largest for professional competence; that through emotional engagement was largest for values and character, interest development, learning ability, and communication and collaboration; and that through cognitive engagement was largest for overall self-evaluation. No single engagement dimension showed the largest specific indirect association across all domains of learning gains. Instead, different engagement dimensions showed distinct patterns of association with different aspects of learning gains. This supports conceptualizing engagement as a multidimensional learning process comprising behavioral, emotional, and cognitive dimensions, rather than as a construct adequately captured by a single indicator.

These findings suggest that learning-experience surveys and evaluations of teaching reforms could assess behavioral, emotional, and cognitive engagement together, rather than relying on behavioral indicators such as study time and frequency of participation, or on a single composite engagement score, to represent students’ learning processes (5). In interpreting monitoring results, the differentiated patterns of the three engagement dimensions across domains of learning gains, considered together with learning environment, can help identify which educational processes merit particular attention. Such multidimensional monitoring can provide process-level information that a single indicator cannot capture and a more differentiated basis for identifying areas for educational improvement across different domains of learning gains.

### 4.3 Stability across survey waves and student heterogeneity beyond average associations

The year-stratified analyses indicated that the main associations between the learning environment and engagement were not confined to any single survey wave. In particular, classroom teaching quality and developmental support were positively associated with behavioral, emotional, and cognitive engagement throughout 2021–2024. The principal environment–engagement associations observed in the pooled sample were therefore consistent across survey waves rather than specific to a particular cohort or year.

Stable average associations, however, do not imply that all students had similar learning experiences. The person-centered analysis revealed marked heterogeneity beneath the average pattern. Notably, the convergent-low profile comprised 37.2% of the sample, with all five indicators approximately 0.60–0.84 SD below their respective sample means. These students were therefore not characterized by a low score in only one aspect of their educational experience; lower learning environment, engagement, and learning gains occurred concurrently. Thus, even when the main associations are stable at the population level, average scores and average associations may conceal a sizeable group of students with consistently less favorable learning experiences.

This heterogeneity was only weakly related to common demographic and family-background characteristics. Sex, rural or urban origin, parental education, household income, and program type were only weakly associated with profile membership (Cramér’s *V* = 0.033–0.063), and the background composition of the convergent-low profile differed only modestly from that of the other profiles. This finding is consistent with the regression results, in which background characteristics explained little of the variance in engagement. Taken together, the variable-centered and person-centered analyses suggest that students’ background characteristics provide limited information about the learning experiences they actually report and cannot substitute for direct measurement of those experiences.

Quality monitoring may therefore need to address not only whether the main associations persist across years but also how students’ experiences are distributed beneath the overall average. Routine learning-experience surveys could be used to identify groups of students with concurrently low scores across several dimensions, rather than relying primarily on demographic or socioeconomic screening. For groups identified in this way, further assessment could consider the specific educational context, such as program, course, and stage of study, while interviews with students in the relevant programs and years could help clarify their learning experiences and support needs. This would extend quality evaluation beyond whether average student experiences appear favorable to identifying which groups report less favorable experiences, providing a more targeted basis for quality improvement.

### 4.4 Implications for educational improvement and innovation

These findings suggest that, in addition to resource allocation and program development, efforts to improve educational quality at medical universities could give greater attention to the teaching and support processes that students experience directly in their everyday learning. The associations of classroom teaching quality and developmental support with engagement provide a basis for considering how existing improvement efforts might be further focused, connected, and evaluated. Firstly, faculty development could place greater emphasis on classroom teaching processes. In this study, classroom teaching quality reflected teachers’ commitment to teaching, classroom organization, and approaches to course assessment; it showed the strongest positive associations with behavioral, emotional, and cognitive engagement in the pooled analysis and was significantly associated with each dimension in every survey wave. This suggests that, in addition to teaching content and the use of technology, faculty development could further address the teaching processes that students experience directly, such as classroom organization, assessment practices, and the quality of feedback (15). Evaluations of faculty development programs could also consider not only whether particular teaching approaches have been implemented, but also how students experience these classroom processes and how they are associated with student engagement.

Secondly, developmental support could be more closely connected with routine educational processes. Developmental support was positively associated with all three engagement dimensions in the pooled analysis, and its coefficients were positive in all year-stratified models. This suggests that existing academic guidance, mentoring, career planning, and developmental opportunities could be more closely linked with coursework, professional development, and stage-specific training tasks, thereby connecting developmental support more closely with students’ everyday learning (11).

Thirdly, student-experience data could be used not only for overall evaluation but also for problem identification and improvement feedback. In this study, profile membership was only weakly associated with the background characteristics examined, suggesting that background characteristics alone may provide limited information for identifying students with less favorable patterns across perceived learning environment, engagement, and learning gains. Routine student-experience surveys could therefore be used not only to describe institution-level conditions but also to provide stratified feedback within specific educational contexts, such as academic programs. Involving students in interpreting the results and designing improvement efforts could further strengthen the use of student-experience data for quality improvement (16).

Fourthly, evaluations of educational innovation could consider both outcomes and learning processes. When teaching reforms or new educational measures are evaluated, process indicators such as perceived learning environment and behavioral, emotional, and cognitive engagement could be considered alongside outcome indicators such as learning gains, with attention to their distribution across student groups rather than overall means alone (5). This approach could support a feedback cycle of identifying problems, implementing adjustments, monitoring learning experiences and engagement, evaluating learning gains, and refining practice, so that evaluation considers not only whether a reform has been implemented but also whether students’ learning experiences and engagement change following its implementation.

### 4.5 Limitations

This study has several limitations. Firstly, the repeated cross-sectional design does not permit conclusions about temporal ordering or causal relationships among learning environment, engagement, and learning gains. The indirect association analysis should therefore be interpreted as a statistical decomposition of contemporaneous associations rather than as evidence of longitudinal mediation (17, 18). Because the survey was anonymous, students who participated in more than one wave could not be identified or linked across years. The analyses therefore describe aggregate patterns across successive survey samples rather than within-student change; repeated participation across waves may also have led to some underestimation of standard errors in the pooled analyses. Longitudinal studies with temporally separated measurements are needed to examine these relationships over time.

Secondly, the numbers of invited or eligible students in each survey wave were not systematically retained. Annual response rates therefore could not be calculated, and non-response bias could not be assessed directly. Whether participants differed systematically from non-participants remains unknown, and systematic differences between respondents and non-respondents cannot be excluded.

Thirdly, learning environment, engagement, and learning gains were all self-reported within the same survey, and the observed associations may therefore have been influenced by common method variance. The survey was anonymous, the constructs were assessed using distinct item content and response formats, and a single-factor diagnostic did not indicate a dominant common component; however, such diagnostics cannot rule out common method bias. Future studies could incorporate complementary data sources, including academic records, course performance, clinical competency assessments, and qualitative data.

Fourthly, the data were obtained from a single medical university in western China and included students from clinical medical, non-clinical medical and health-related, and non-medical programs. The findings therefore primarily reflect undergraduate learning experiences within this institutional context, and their generalizability to other regions, other types of institutions, and specifically to students in clinical medical programs requires further examination.

Fifthly, the k-means profiles describe the joint distribution of learning environment, engagement, and learning gains in the present sample rather than stable or enduring student types. The three-profile solution showed only modest separation, and the proportions assigned to each profile depend partly on the number of clusters specified. The profiles should therefore be regarded as an exploratory representation of student heterogeneity rather than as discrete groups with clear boundaries. Their replicability and longitudinal stability should be examined in independent samples, together with their relationships with objective educational outcomes.

## 5 Conclusion

For quality improvement in medical education, these findings suggest that attention should extend beyond resource provision alone to the teaching and developmental support processes that students experience directly. Routine monitoring could also incorporate multidimensional engagement alongside students’ own reports of their learning experiences, helping to identify groups with less favorable experiences that are not readily distinguished by background characteristics. Whether improvements in these processes are followed by greater engagement and learning gains remains to be established in prospective and intervention studies.

## Data Availability

The datasets analyzed in this study are not publicly available because they are subject to institutional data-access restrictions. De-identified data are available upon reasonable request. Aggregated results are provided in the article and Supplementary Tables S1–S8. Requests for data access should be directed to the corresponding authors.
