# Supplementary material for: Evaluating the associations of learning environment with student engagement and learning gains among undergraduate students at a medical university in western China
Source: Front Public Health. 2026 Oct 19;14:1885067. doi: 10.3389/fpubh.2026.1885067 (PMC13724328; doi:10.3389/fpubh.2026.1885067)
Supplement: Supplementary file 1 [file Data_Sheet_1.docx]

**Supplementary Table S1. Between-year differences in cohort composition and core study variables across four NCSS administrations, 2021–2024 (*N* = 11,717)**

| **Section** | **Variable** | **Category** | **2021**  **(*N* = 1,015)** | **2022**  **(*N* = 4,222)** | **2023**  **(*N* = 3,028)** | **2024**  **(*N* = 3,452)** | **Statistic** | ***p*** | **Effect size** | **Magnitude** |
| --- | --- | --- | --- | --- | --- | --- | --- | --- | --- | --- |
| **Panel A**  **Background characteristics — *N* (%); χ²; Cramé*r*’s *V*** | | | | | | | | | | |
| Background characteristics | Sex | Male | 359 (35.4%) | 1,404 (33.3%) | 1,021 (33.7%) | 943 (27.3%) | 47.19 | < .001 | 0.063 | Negligible |
|  |  | Female | 656 (64.6%) | 2,818 (66.7%) | 2,007 (66.3%) | 2,509 (72.7%) |  |  |  |  |
|  | Year of study | Year 1 | 265 (26.1%) | 1,414 (33.5%) | 915 (30.2%) | 1,183 (34.3%) | 410.09 | < .001 | 0.108 | Small |
|  |  | Year 2 | 269 (26.5%) | 1,355 (32.1%) | 865 (28.6%) | 847 (24.5%) |  |  |  |  |
|  |  | Year 3 | 263 (25.9%) | 1,214 (28.8%) | 880 (29.1%) | 958 (27.8%) |  |  |  |  |
|  |  | Year 4 | 147 (14.5%) | 168 (4.0%) | 346 (11.4%) | 391 (11.3%) |  |  |  |  |
|  |  | Year 5 | 71 (7.0%) | 71 (1.7%) | 22 (0.7%) | 73 (2.1%) |  |  |  |  |
|  | Ethnicity | Han | 677 (66.7%) | 2,891 (68.5%) | 2,091 (69.1%) | 2,288 (66.3%) | 7.28 | .063 | 0.025 | Negligible |
|  |  | Ethnic minority | 338 (33.3%) | 1,331 (31.5%) | 937 (30.9%) | 1,164 (33.7%) |  |  |  |  |
|  | Rural/urban origin | Urban | 298 (29.4%) | 1,319 (31.2%) | 824 (27.2%) | 1,056 (30.6%) | 14.98 | .002 | 0.036 | Negligible |
|  |  | Rural | 717 (70.6%) | 2,903 (68.8%) | 2,204 (72.8%) | 2,396 (69.4%) |  |  |  |  |
|  | Parental education | High school or below | 799 (78.7%) | 3,310 (78.4%) | 2,400 (79.3%) | 2,787 (80.7%) | 6.63 | .085 | 0.024 | Negligible |
|  |  | Junior college or above | 216 (21.3%) | 912 (21.6%) | 628 (20.7%) | 665 (19.3%) |  |  |  |  |
|  | Household income | Low | 687 (67.7%) | 2,831 (67.1%) | 2,007 (66.3%) | 2,222 (64.4%) | 8.30 | .217 | 0.019 | Negligible |
|  |  | Middle | 262 (25.8%) | 1,135 (26.9%) | 832 (27.5%) | 1,009 (29.2%) |  |  |  |  |
|  |  | High | 66 (6.5%) | 256 (6.1%) | 189 (6.2%) | 221 (6.4%) |  |  |  |  |
|  | Program type | Clinical medical | 578 (56.9%) | 2,284 (54.1%) | 1,643 (54.3%) | 1,037 (30.0%) | 638.59 | < .001 | 0.165 | Small |
|  |  | Non-clinical medical and health-related | 378 (37.2%) | 1,438 (34.1%) | 1,158 (38.2%) | 1,893 (54.8%) |  |  |  |  |
|  |  | Non-medical | 59 (5.8%) | 500 (11.8%) | 227 (7.5%) | 522 (15.1%) |  |  |  |  |
| **Panel B**  **Core study variables — M (*SD*); one-way ANOVA *F*; η²** | | | | | | | | | | |
| Composite scores | Perceived learning environment |  | 4.47 (0.59) | 4.44 (0.62) | 4.47 (0.64) | 4.55 (0.65) | 18.79 | < .001 | 0.0048 | Negligible |
|  | Learning gains |  | 4.55 (0.64) | 4.40 (0.67) | 4.39 (0.68) | 4.47 (0.68) | 21.42 | < .001 | 0.0055 | Negligible |
| Perceived learning environment | Classroom teaching quality |  | 4.39 (0.55) | 4.42 (0.56) | 4.43 (0.58) | 4.50 (0.58) | 17.38 | < .001 | 0.0044 | Negligible |
|  | Facilities and resources |  | 4.38 (0.73) | 4.30 (0.78) | 4.37 (0.78) | 4.47 (0.79) | 30.33 | < .001 | 0.0077 | Negligible |
|  | Developmental support |  | 4.43 (0.64) | 4.40 (0.67) | 4.44 (0.70) | 4.51 (0.69) | 18.14 | < .001 | 0.0046 | Negligible |
|  | Interpersonal support |  | 4.65 (0.69) | 4.62 (0.71) | 4.62 (0.73) | 4.69 (0.74) | 7.57 | < .001 | 0.0019 | Negligible |
|  | Teaching administration |  | 4.48 (0.76) | 4.48 (0.76) | 4.47 (0.79) | 4.57 (0.79) | 11.22 | < .001 | 0.0029 | Negligible |
| Student engagement | Behavioral engagement |  | 3.16 (0.53) | 3.17 (0.59) | 3.20 (0.61) | 3.26 (0.59) | 18.67 | < .001 | 0.0048 | Negligible |
|  | Emotional engagement |  | 4.60 (0.60) | 4.52 (0.61) | 4.44 (0.62) | 4.47 (0.62) | 21.37 | < .001 | 0.0054 | Negligible |
|  | Cognitive engagement |  | 3.94 (0.52) | 3.88 (0.52) | 3.88 (0.53) | 3.94 (0.54) | 10.72 | < .001 | 0.0027 | Negligible |
| Learning gains | Professional competence |  | 4.41 (0.70) | 4.22 (0.72) | 4.23 (0.73) | 4.31 (0.72) | 25.58 | < .001 | 0.0065 | Negligible |
|  | Values and character |  | 4.66 (0.71) | 4.49 (0.74) | 4.47 (0.76) | 4.52 (0.75) | 16.30 | < .001 | 0.0042 | Negligible |
|  | Interest development |  | 4.55 (0.71) | 4.38 (0.74) | 4.37 (0.75) | 4.44 (0.73) | 20.28 | < .001 | 0.0052 | Negligible |
|  | Learning ability |  | 4.50 (0.65) | 4.37 (0.70) | 4.34 (0.70) | 4.43 (0.71) | 17.84 | < .001 | 0.0045 | Negligible |
|  | Overall self-evaluation |  | 4.49 (0.78) | 4.44 (0.86) | 4.44 (0.86) | 4.58 (0.85) | 20.62 | < .001 | 0.0053 | Negligible |
|  | Communication and collaboration |  | 4.67 (0.74) | 4.47 (0.83) | 4.46 (0.83) | 4.52 (0.80) | 19.26 | < .001 | 0.0049 | Negligible |

**Notes**: Panel A reports column percentages and Pearson χ² statistics with Cramér’s V for cohort-composition variables. Panel B reports mean (SD), one-way ANOVA F statistics, and η² for core study variables. Magnitude labels were defined as negligible (<0.01 for η²; <0.10 for V), small (0.01 to <0.06 for η²; 0.10 to <0.30 for V), moderate (0.06 to <0.14 for η²; 0.30 to <0.50 for V), and large (≥0.14 for η²; ≥0.50 for V). η² values are reported to four decimal places to preserve precision for very small effects.

**Supplementary Table S2. Descriptive statistics and internal consistency of study measures (items scored 1–6)**

| **Construct** | **Subscale** | **Items** | **Cronbach’s α** | **Mean (SD)** |
| --- | --- | --- | --- | --- |
| learning environment | Classroom teaching quality | 19 | 0.93 | 4.44 (0.57) |
|  | Facilities and resources | 10 | 0.90 | 4.37 (0.78) |
|  | Developmental support | 9 | 0.89 | 4.45 (0.68) |
|  | Interpersonal support | 6 | 0.91 | 4.64 (0.73) |
|  | Teaching administration | 4 | 0.84 | 4.50 (0.78) |
| Student engagement | Behavioral engagement | 32 | 0.90 | 3.20 (0.59) |
|  | Emotional engagement | 13 | 0.86 | 4.49 (0.62) |
|  | Cognitive engagement | 19 | 0.84 | 3.90 (0.53) |
| Learning gains | Professional competence | 5 | 0.88 | 4.26 (0.72) |
|  | Values and character | 5 | 0.91 | 4.51 (0.75) |
|  | Interest development | 5 | 0.90 | 4.41 (0.74) |
|  | Learning ability | 9 | 0.93 | 4.39 (0.70) |
|  | Communication and collaboration | 3 | 0.93 | 4.50 (0.82) |
|  | Overall self-evaluation | 2 | 0.72 | 4.49 (0.85) |

**Notes**: Scale scores are means of applicable items. For eight 2021 student responses, the daily English-study-time item was not applicable and was treated as missing; behavioral engagement scores were calculated from the remaining 31 items. Cronbach’s α for behavioral engagement was therefore based on 11,709 complete cases; all other statistics used 11,717 student responses.

**Supplementary Table S3. Group differences in student engagement, perceived learning environment, and learning gains by background characteristics (*N* = 11,717)**

| **Characteristic** | **Category** | **Overall engagement** | | | **Perceived learning environment** | | | **Learning gains** | | |
| --- | --- | --- | --- | --- | --- | --- | --- | --- | --- | --- |
|  |  | **mean (*SD*)** | ***p*** | **Effect size** | **mean (*SD*)** | ***p*** | **Effect size** | **mean (*SD*)** | ***p*** | **Effect size** |
| Sex | Male | 3.83 (0.51) | < .001 | *d* = 0.110 | 4.48 (0.69) | .519 | *d* = 0.013 | 4.41 (0.74) | .098 | *d* = 0.035 |
|  | Female | 3.88 (0.45) |  |  | 4.48 (0.60) |  |  | 4.43 (0.64) |  |  |
| Ethnicity | Han | 3.88 (0.47) | < .001 | *d* = −0.066 | 4.48 (0.63) | .633 | *d* = −0.009 | 4.44 (0.67) | .029 | *d* = −0.043 |
|  | Ethnic minority | 3.85 (0.47) |  |  | 4.48 (0.63) |  |  | 4.41 (0.68) |  |  |
| Rural/urban origin | Urban | 3.89 (0.51) | < .001 | *d* = −0.087 | 4.48 (0.69) | .588 | *d* = 0.011 | 4.45 (0.74) | .032 | *d* = −0.046 |
|  | Rural | 3.85 (0.45) |  |  | 4.48 (0.61) |  |  | 4.42 (0.64) |  |  |
| Parental education | High school or below | 3.85 (0.46) | < .001 | *d* = 0.131 | 4.48 (0.62) | .320 | *d* = 0.024 | 4.42 (0.65) | .004 | *d* = 0.071 |
|  | Junior college or above | 3.91 (0.51) |  |  | 4.49 (0.69) |  |  | 4.46 (0.75) |  |  |
| Year of study | Year 1 | 3.83 (0.47) | < .001 | η² = 0.0031 | 4.54 (0.63) | < .001 | η² = 0.0052 | 4.42 (0.66) | .329 | η² = 0.0004 |
|  | Year 2 | 3.88 (0.47) |  |  | 4.48 (0.63) |  |  | 4.43 (0.68) |  |  |
|  | Year 3 | 3.88 (0.47) |  |  | 4.43 (0.64) |  |  | 4.42 (0.68) |  |  |
|  | Year 4 | 3.90 (0.47) |  |  | 4.46 (0.63) |  |  | 4.45 (0.65) |  |  |
|  | Year 5 | 3.93 (0.43) |  |  | 4.48 (0.59) |  |  | 4.48 (0.65) |  |  |
| Program type | Clinical medical | 3.88 (0.48) | < .001 | η² = 0.0015 | 4.49 (0.64) | < .001 | η² = 0.0022 | 4.44 (0.68) | < .001 | η² = 0.0033 |
|  | Non-clinical medical and health-related | 3.86 (0.46) |  |  | 4.49 (0.63) |  |  | 4.44 (0.66) |  |  |
|  | Non-medical | 3.82 (0.45) |  |  | 4.40 (0.62) |  |  | 4.32 (0.67) |  |  |
| Household income | Low | 3.84 (0.46) | < .001 | η² = 0.0095 | 4.46 (0.62) | < .001 | η² = 0.0031 | 4.39 (0.65) | < .001 | η² = 0.0070 |
|  | Middle | 3.89 (0.47) |  |  | 4.52 (0.64) |  |  | 4.47 (0.69) |  |  |
|  | High | 4.02 (0.52) |  |  | 4.56 (0.72) |  |  | 4.60 (0.76) |  |  |
| Survey year | 2021 | 3.90 (0.44) | < .001 | η² = 0.0020 | 4.47 (0.59) | < .001 | η² = 0.0048 | 4.55 (0.64) | < .001 | η² = 0.0055 |
|  | 2022 | 3.86 (0.46) |  |  | 4.44 (0.62) |  |  | 4.40 (0.67) |  |  |
|  | 2023 | 3.84 (0.48) |  |  | 4.47 (0.64) |  |  | 4.39 (0.68) |  |  |
|  | 2024 | 3.89 (0.48) |  |  | 4.55 (0.65) |  |  | 4.47 (0.68) |  |  |

**Notes**: Values are mean (*SD*) unless otherwise indicated. *p* values are from Welch’s *t* tests for two-group variables and one-way ANOVA for variables with three or more groups. For every two-group comparison, Cohen’s *d* was calculated as the mean of the second listed category minus the mean of the first listed category, divided by the pooled *SD*; negative values therefore indicate a lower mean in the second listed category. η² is reported for comparisons involving three or more groups. η² values are reported to four decimal places to preserve precision for very small effects.

**Supplementary Table S4. Specific indirect associations of each perceived learning environment domain with overall learning gains through the three engagement dimensions (standardized estimates)**

| **Learning environment dimension** | **Behavioral engagement** | **Emotional engagement** | **Cognitive engagement** |
| --- | --- | --- | --- |
| Classroom teaching quality | 0.074^***^ (0.064, 0.084) | 0.095^***^ (0.084, 0.105) | 0.086^***^ (0.076, 0.096) |
| Facilities and resources | 0.078^***^ (0.069, 0.086) | 0.099^***^ (0.091, 0.108) | 0.061^***^ (0.053, 0.070) |
| Developmental support | 0.076^***^ (0.067, 0.085) | 0.097^***^ (0.087, 0.106) | 0.075^***^ (0.066, 0.085) |
| Interpersonal support | 0.077^***^ (0.068, 0.085) | 0.097^***^ (0.088, 0.106) | 0.068^***^ (0.060, 0.077) |
| Teaching administration | 0.074^***^ (0.066, 0.082) | 0.098^***^ (0.089, 0.107) | 0.063^***^ (0.054, 0.071) |

**Notes**: Values are standardized specific indirect associations (95% percentile bootstrap CI) for the path environment domain → engagement dimension → overall learning gains, adjusted for survey year, sex, year of study, ethnicity, rural/urban origin, parental education, household income, and program type.

Confidence intervals are percentile intervals from 10,000 bootstrap resamples; intervals excluding zero indicate a nonzero indirect association.

^***^*p* < .001.

**Supplementary Table S5. Specific indirect associations of the overall perceived learning environment with domain-specific learning gains through behavioral, emotional, and cognitive engagement (standardized estimates)**

| **Learning-gains domain** | **Behavioral engagement** | **Emotional engagement** | **Cognitive engagement** |
| --- | --- | --- | --- |
| Professional competence | 0.093^***^ (0.082, 0.103) | 0.050^***^ (0.040, 0.060) | 0.070^***^ (0.059, 0.081) |
| Values and character | 0.053^***^ (0.043, 0.062) | 0.121^***^ (0.110, 0.132) | 0.051^***^ (0.041, 0.062) |
| Interest development | 0.077^***^ (0.068, 0.087) | 0.103^***^ (0.092, 0.114) | 0.067^***^ (0.057, 0.078) |
| Learning ability | 0.070^***^ (0.061, 0.079) | 0.098^***^ (0.087, 0.108) | 0.084^***^ (0.074, 0.095) |
| Communication and collaboration | 0.066^***^ (0.056, 0.075) | 0.103^***^ (0.091, 0.114) | 0.051^***^ (0.040, 0.063) |
| Overall self-evaluation | 0.036^***^ (0.028, 0.043) | 0.006 (−0.003, 0.015) | 0.060^***^ (0.050, 0.069) |

**Notes**: Values are standardized specific indirect associations (95% percentile bootstrap CI, 10,000 resamples) for the path overall learning environment → engagement dimension → learning-gains domain, adjusted for the same covariates as in Supplementary Table S4. ^***^*p* < .001; the estimate without asterisks has a 95% CI that includes zero.

**Supplementary Table S6. Summary of tested interaction effects on student engagement outcomes**

| **Interaction term** | **Outcome (dependent variable)** | ***F*** | ***p*** |
| --- | --- | --- | --- |
| Classroom teaching quality × Year of study | Behavioral engagement | 1.13 | .341 |
| Classroom teaching quality × Year of study | Emotional engagement | 1.85 | .115 |
| Classroom teaching quality × Year of study | Cognitive engagement | 0.73 | .569 |
| Interpersonal support × Program type | Emotional engagement | 1.60 | .201 |
| Developmental support × Parental education | Cognitive engagement | 3.02 | .082 |
| Overall perceived learning environment × Sex | Overall engagement | 0.16 | .687 |

**Notes**: Interaction effects were tested by adding product terms to the adjusted regression models. *F* statistics correspond to the omnibus test of the interaction term(s). *p* values are two-sided and unadjusted for multiple testing. No interaction reached nominal significance (all *p* > .05), and none met the Bonferroni-corrected threshold for six tests (α = 0.0083).

**Supplementary Table S7. Year-stratified regression coefficients for associations between perceived learning environment domains and student engagement across the four survey waves**

| **Year** | ***N*** | **Outcome** | **Predictor** | ***B*** | ***β*** | **OLS *SE*** | **OLS *p*** | **OLS 95% CI** | **HC3 *SE*** | **HC3 *p*** | **HC3 95% CI** |
| --- | --- | --- | --- | --- | --- | --- | --- | --- | --- | --- | --- |
| 2021 | 1,015 | Behavioral engagement | Classroom teaching quality | 0.352 | 0.362 | 0.045 | < .001 | 0.263, 0.441 | 0.063 | < .001 | 0.228, 0.477 |
| 2021 | 1,015 | Behavioral engagement | Facilities and resources | −0.070 | −0.097 | 0.037 | .058 | −0.143, 0.002 | 0.042 | .095 | −0.153, 0.012 |
| 2021 | 1,015 | Behavioral engagement | Developmental support | 0.139 | 0.168 | 0.048 | .004 | 0.045, 0.232 | 0.061 | .024 | 0.018, 0.259 |
| 2021 | 1,015 | Behavioral engagement | Interpersonal support | 0.005 | 0.006 | 0.036 | .894 | −0.066, 0.076 | 0.044 | .912 | −0.081, 0.090 |
| 2021 | 1,015 | Behavioral engagement | Teaching administration | −0.047 | −0.068 | 0.035 | .174 | −0.115, 0.021 | 0.045 | .294 | −0.135, 0.041 |
| 2021 | 1,015 | Emotional engagement | Classroom teaching quality | 0.458 | 0.419 | 0.047 | < .001 | 0.365, 0.550 | 0.059 | < .001 | 0.342, 0.574 |
| 2021 | 1,015 | Emotional engagement | Facilities and resources | −0.152 | −0.186 | 0.039 | < .001 | −0.227, −0.076 | 0.042 | < .001 | −0.234, −0.070 |
| 2021 | 1,015 | Emotional engagement | Developmental support | 0.099 | 0.107 | 0.050 | .045 | 0.002, 0.197 | 0.058 | .085 | −0.014, 0.213 |
| 2021 | 1,015 | Emotional engagement | Interpersonal support | 0.123 | 0.143 | 0.038 | .001 | 0.049, 0.197 | 0.049 | .012 | 0.027, 0.219 |
| 2021 | 1,015 | Emotional engagement | Teaching administration | 0.083 | 0.106 | 0.036 | .022 | 0.012, 0.153 | 0.051 | .107 | −0.018, 0.183 |
| 2021 | 1,015 | Cognitive engagement | Classroom teaching quality | 0.357 | 0.378 | 0.044 | < .001 | 0.271, 0.443 | 0.063 | < .001 | 0.233, 0.481 |
| 2021 | 1,015 | Cognitive engagement | Facilities and resources | −0.036 | −0.051 | 0.036 | .314 | −0.106, 0.034 | 0.041 | .376 | −0.116, 0.044 |
| 2021 | 1,015 | Cognitive engagement | Developmental support | 0.039 | 0.049 | 0.046 | .396 | −0.051, 0.129 | 0.064 | .545 | −0.087, 0.166 |
| 2021 | 1,015 | Cognitive engagement | Interpersonal support | 0.036 | 0.049 | 0.035 | .301 | −0.033, 0.105 | 0.045 | .422 | −0.052, 0.125 |
| 2021 | 1,015 | Cognitive engagement | Teaching administration | 0.032 | 0.047 | 0.033 | .343 | −0.034, 0.097 | 0.053 | .549 | −0.072, 0.135 |
| 2022 | 4,222 | Behavioral engagement | Classroom teaching quality | 0.369 | 0.348 | 0.023 | < .001 | 0.323, 0.414 | 0.028 | < .001 | 0.313, 0.424 |
| 2022 | 4,222 | Behavioral engagement | Facilities and resources | 0.026 | 0.034 | 0.018 | .149 | −0.009, 0.061 | 0.020 | .189 | −0.013, 0.064 |
| 2022 | 4,222 | Behavioral engagement | Developmental support | 0.210 | 0.236 | 0.025 | < .001 | 0.161, 0.258 | 0.028 | < .001 | 0.154, 0.265 |
| 2022 | 4,222 | Behavioral engagement | Interpersonal support | 0.010 | 0.012 | 0.019 | .592 | −0.027, 0.047 | 0.021 | .637 | −0.032, 0.052 |
| 2022 | 4,222 | Behavioral engagement | Teaching administration | −0.115 | −0.147 | 0.020 | < .001 | −0.154, −0.076 | 0.023 | < .001 | −0.160, −0.070 |
| 2022 | 4,222 | Emotional engagement | Classroom teaching quality | 0.377 | 0.344 | 0.024 | < .001 | 0.330, 0.423 | 0.030 | < .001 | 0.317, 0.436 |
| 2022 | 4,222 | Emotional engagement | Facilities and resources | −0.104 | −0.133 | 0.018 | < .001 | −0.140, −0.069 | 0.021 | < .001 | −0.146, −0.062 |
| 2022 | 4,222 | Emotional engagement | Developmental support | 0.112 | 0.122 | 0.025 | < .001 | 0.063, 0.161 | 0.031 | < .001 | 0.052, 0.172 |
| 2022 | 4,222 | Emotional engagement | Interpersonal support | 0.130 | 0.150 | 0.019 | < .001 | 0.092, 0.167 | 0.023 | < .001 | 0.084, 0.175 |
| 2022 | 4,222 | Emotional engagement | Teaching administration | 0.040 | 0.049 | 0.020 | .048 | 0.000, 0.079 | 0.024 | .101 | −0.008, 0.087 |
| 2022 | 4,222 | Cognitive engagement | Classroom teaching quality | 0.257 | 0.275 | 0.021 | < .001 | 0.215, 0.298 | 0.027 | < .001 | 0.204, 0.309 |
| 2022 | 4,222 | Cognitive engagement | Facilities and resources | 0.022 | 0.033 | 0.016 | .175 | −0.010, 0.053 | 0.019 | .250 | −0.015, 0.059 |
| 2022 | 4,222 | Cognitive engagement | Developmental support | 0.131 | 0.167 | 0.022 | < .001 | 0.087, 0.174 | 0.028 | < .001 | 0.076, 0.186 |
| 2022 | 4,222 | Cognitive engagement | Interpersonal support | 0.074 | 0.100 | 0.017 | < .001 | 0.040, 0.107 | 0.020 | < .001 | 0.035, 0.112 |
| 2022 | 4,222 | Cognitive engagement | Teaching administration | −0.041 | −0.060 | 0.018 | .020 | −0.076, −0.006 | 0.022 | .059 | −0.084, 0.002 |
| 2023 | 3,028 | Behavioral engagement | Classroom teaching quality | 0.340 | 0.324 | 0.028 | < .001 | 0.285, 0.395 | 0.037 | < .001 | 0.267, 0.412 |
| 2023 | 3,028 | Behavioral engagement | Facilities and resources | 0.019 | 0.024 | 0.023 | .414 | −0.027, 0.064 | 0.026 | .472 | −0.033, 0.070 |
| 2023 | 3,028 | Behavioral engagement | Developmental support | 0.196 | 0.225 | 0.030 | < .001 | 0.136, 0.255 | 0.036 | < .001 | 0.125, 0.266 |
| 2023 | 3,028 | Behavioral engagement | Interpersonal support | 0.023 | 0.027 | 0.022 | .303 | −0.021, 0.066 | 0.028 | .418 | −0.032, 0.078 |
| 2023 | 3,028 | Behavioral engagement | Teaching administration | −0.080 | −0.104 | 0.023 | < .001 | −0.125, −0.035 | 0.027 | .003 | −0.132, −0.027 |
| 2023 | 3,028 | Emotional engagement | Classroom teaching quality | 0.289 | 0.269 | 0.028 | < .001 | 0.234, 0.344 | 0.036 | < .001 | 0.218, 0.360 |
| 2023 | 3,028 | Emotional engagement | Facilities and resources | −0.092 | −0.115 | 0.023 | < .001 | −0.138, −0.047 | 0.026 | < .001 | −0.143, −0.042 |
| 2023 | 3,028 | Emotional engagement | Developmental support | 0.217 | 0.244 | 0.030 | < .001 | 0.158, 0.276 | 0.038 | < .001 | 0.143, 0.292 |
| 2023 | 3,028 | Emotional engagement | Interpersonal support | 0.098 | 0.116 | 0.022 | < .001 | 0.055, 0.142 | 0.028 | < .001 | 0.044, 0.153 |
| 2023 | 3,028 | Emotional engagement | Teaching administration | 0.024 | 0.031 | 0.023 | .288 | −0.020, 0.069 | 0.027 | .379 | −0.030, 0.078 |
| 2023 | 3,028 | Cognitive engagement | Classroom teaching quality | 0.192 | 0.210 | 0.025 | < .001 | 0.143, 0.240 | 0.030 | < .001 | 0.133, 0.251 |
| 2023 | 3,028 | Cognitive engagement | Facilities and resources | 0.006 | 0.008 | 0.020 | .781 | −0.034, 0.046 | 0.023 | .808 | −0.040, 0.052 |
| 2023 | 3,028 | Cognitive engagement | Developmental support | 0.173 | 0.229 | 0.027 | < .001 | 0.121, 0.226 | 0.032 | < .001 | 0.110, 0.237 |
| 2023 | 3,028 | Cognitive engagement | Interpersonal support | 0.063 | 0.087 | 0.020 | .001 | 0.025, 0.101 | 0.026 | .017 | 0.011, 0.114 |
| 2023 | 3,028 | Cognitive engagement | Teaching administration | −0.018 | −0.027 | 0.020 | .378 | −0.057, 0.022 | 0.023 | .445 | −0.063, 0.028 |
| 2024 | 3,452 | Behavioral engagement | Classroom teaching quality | 0.397 | 0.387 | 0.027 | < .001 | 0.344, 0.449 | 0.032 | < .001 | 0.334, 0.459 |
| 2024 | 3,452 | Behavioral engagement | Facilities and resources | −0.006 | −0.008 | 0.021 | .785 | −0.048, 0.036 | 0.023 | .804 | −0.052, 0.040 |
| 2024 | 3,452 | Behavioral engagement | Developmental support | 0.218 | 0.255 | 0.028 | < .001 | 0.163, 0.273 | 0.034 | < .001 | 0.151, 0.284 |
| 2024 | 3,452 | Behavioral engagement | Interpersonal support | −0.013 | −0.016 | 0.021 | .549 | −0.054, 0.029 | 0.026 | .623 | −0.063, 0.038 |
| 2024 | 3,452 | Behavioral engagement | Teaching administration | −0.106 | −0.142 | 0.022 | < .001 | −0.149, −0.063 | 0.026 | < .001 | −0.158, −0.054 |
| 2024 | 3,452 | Emotional engagement | Classroom teaching quality | 0.450 | 0.420 | 0.027 | < .001 | 0.397, 0.504 | 0.031 | < .001 | 0.390, 0.510 |
| 2024 | 3,452 | Emotional engagement | Facilities and resources | −0.060 | −0.077 | 0.022 | .006 | −0.102, −0.017 | 0.024 | .014 | −0.107, −0.012 |
| 2024 | 3,452 | Emotional engagement | Developmental support | 0.131 | 0.147 | 0.028 | < .001 | 0.076, 0.187 | 0.031 | < .001 | 0.071, 0.192 |
| 2024 | 3,452 | Emotional engagement | Interpersonal support | 0.064 | 0.077 | 0.021 | .003 | 0.022, 0.106 | 0.024 | .007 | 0.017, 0.111 |
| 2024 | 3,452 | Emotional engagement | Teaching administration | 0.012 | 0.016 | 0.022 | .584 | −0.032, 0.056 | 0.025 | .625 | −0.037, 0.062 |
| 2024 | 3,452 | Cognitive engagement | Classroom teaching quality | 0.330 | 0.351 | 0.025 | < .001 | 0.281, 0.379 | 0.029 | < .001 | 0.273, 0.387 |
| 2024 | 3,452 | Cognitive engagement | Facilities and resources | 0.000 | 0.000 | 0.020 | .997 | −0.038, 0.039 | 0.022 | .997 | −0.043, 0.043 |
| 2024 | 3,452 | Cognitive engagement | Developmental support | 0.141 | 0.180 | 0.026 | < .001 | 0.091, 0.192 | 0.032 | < .001 | 0.079, 0.203 |
| 2024 | 3,452 | Cognitive engagement | Interpersonal support | 0.039 | 0.054 | 0.020 | .043 | 0.001, 0.078 | 0.024 | .095 | −0.007, 0.086 |
| 2024 | 3,452 | Cognitive engagement | Teaching administration | −0.027 | −0.039 | 0.020 | .183 | −0.067, 0.013 | 0.024 | .258 | −0.074, 0.020 |

**Notes**: Results are from the full year-stratified multiple linear regression models. Each model adjusted for sex, year of study, ethnicity, rural/urban origin, parental education, household income, and program type, and included the five perceived learning environment domains. *B* indicates unstandardized regression coefficients; β indicates standardized regression coefficients. OLS columns use conventional model-based standard errors, *p* values, and 95% confidence intervals; HC3 columns use heteroskedasticity-consistent standard errors, *p* values, and 95% confidence intervals. Confidence intervals refer to *B*.

**Supplementary Table S8. Academic-program classification based on training objectives, curriculum structure, and practical-training requirements (N = 11,717)**

| **Program type** | **Academic program** | **Primary training focus** | **N** |
| --- | --- | --- | --- |
| Clinical medical programs (N = 5,542) | Clinical Medicine | Clinical diagnosis and physician training | 2,403 |
|  | Clinical Medicine (state-funded, rural-oriented) | Clinical diagnosis and physician training | 1,047 |
|  | Clinical Medicine (5+3 integrated) | Clinical diagnosis and physician training | 767 |
|  | Pediatrics | Pediatric clinical care | 279 |
|  | Stomatology | Oral diagnosis and treatment | 276 |
|  | Maternal and Child Health Medicine | Maternal and child clinical care | 190 |
|  | Medical Imaging | Clinical imaging and diagnosis | 140 |
|  | Anesthesiology | Anesthesia and perioperative care | 131 |
|  | Clinical Medicine (rural-oriented, contracted) | Clinical diagnosis and physician training | 131 |
|  | Psychiatry | Psychiatric diagnosis and treatment | 118 |
|  | Clinical Medicine (5+3 integrated, bilingual) | Clinical diagnosis and physician training | 27 |
|  | Clinical Medicine (bilingual) | Clinical diagnosis and physician training | 15 |
|  | Clinical Medicine (curriculum-reform class) | Clinical diagnosis and physician training | 12 |
|  | Clinical Medicine (5+3 integrated, pediatrics) | Pediatric clinical care | 6 |
| Non-clinical medical and health-related programs (N = 4,867) | Nursing | Nursing care | 1,617 |
|  | Pharmacy | Pharmaceutical sciences and practice | 648 |
|  | Preventive Medicine | Public health and disease prevention | 605 |
|  | Clinical Pharmacy | Clinical pharmacy | 533 |
|  | Sports Rehabilitation | Rehabilitation and exercise practice | 241 |
|  | Health Inspection and Quarantine | Public-health laboratory practice | 182 |
|  | Medical Information Engineering | Health-information engineering | 148 |
|  | Health Services and Management | Health services and management | 130 |
|  | Rehabilitation Therapy | Rehabilitation therapy | 110 |
|  | Biomedical Engineering | Biomedical engineering | 109 |
|  | Midwifery | Midwifery care | 91 |
|  | Medical Experimental Technology | Medical laboratory and experimental technology | 80 |
|  | Biotechnology | Biomedical and life sciences | 75 |
|  | Forensic Medicine | Forensic medical science | 73 |
|  | Medical Laboratory Technology | Medical laboratory technology | 64 |
|  | Chinese Materia Medica Resources and Development | Chinese materia medica | 62 |
|  | Chinese Materia Medica | Chinese materia medica | 44 |
|  | Nursing (Yulin campus) | Nursing care | 31 |
|  | Intelligent Medical Engineering | Intelligent medical technology | 14 |
|  | Pharmacy (Yulin campus) | Pharmaceutical sciences and practice | 10 |
| Non-medical programs (N = 1,308) | Information Management and Information Systems | Information systems and management | 278 |
|  | Public Administration | Public administration | 237 |
|  | Social Work | Social services | 222 |
|  | English | Language studies | 181 |
|  | Elderly Care Services Management | Elderly-care management | 149 |
|  | Public Administration (social medical security) | Public administration | 93 |
|  | Translation | Language studies | 87 |
|  | Public Administration (health administration) | Public administration | 37 |
|  | Public Administration (joint program) | Public administration | 16 |
|  | Medical Insurance | Insurance and public administration | 8 |

**Notes**: Programs were grouped according to similarities in their training objectives, curriculum structure, and practical-training requirements.
